# Developing and optimizing an equitable and mutually beneficial virtual global health education partnership

**DOI:** 10.5116/ijme.6563.1b81

**Published:** 2023-12-22

**Authors:** Bianca Nfonoyim, Madeline Chandra, Betsy Bate, Karen Ekotto, Emmanuel Jongwane, Lucie Ngaba, Pierre Ngaba, Vera Njock, Leonel Toledo, Andrew Steenhoff, Morgan Congdon, Charlotte Ekoube

**Affiliations:** 1 Department of Paediatrics & Global Health Centre, The Children's Hospital of Philadelphia, Philadelphia, PA, United States; 2 2Department of Paediatrics, Laquintinie Hospital in Douala, Douala, Cameroon; 3 Department of Paediatrics, Laquintinie Hospital in Douala, Douala, Cameroon; 4 Division of Neurology, The Children's Hospital of Philadelphia, Philadelphia, PA, United States; 5 Citizen Diplomacy International, Philadelphia, PA, United States

**Keywords:** Global health education, virtual global health, health education partnership, general paediatrics GHE partnership

## Introduction

Interest in global health in academia has increased, with institutions worldwide now participating in global health education (GHE) partnerships.^1^ These have allowed trainees and physicians in low and middle-income countries (LMIC) and high-income countries (HIC) to benefit from education, mentorship, and career development opportunities. Further, effective, bi-directional GHE partnerships enhance cross-cultural communication and afford unique learning experiences for all participants. Despite these benefits, many global partnerships have inadvertently contributed to power imbalances, with the agenda, research, and funding opportunities being driven by HIC.^2^^,^^3^^,^^4^^,^^5^

The disruption of global health activities during the COVID-19 pandemic provided a timely pause to examine factors contributing to differential benefits in GHE partnerships, such as lack of shared vision and insufficient evaluation and quality improvement (QI) practices.^6^ This article highlights potential barriers and strategies to create and continually improve an effective GHE partnership.

### Approach

The goal of our project is to promote a mutually beneficial general paediatrics GHE partnership between Laquintinie Hospital in Douala, Cameroon (LHD) and the Children's Hospital of Philadelphia (CHOP), United States, the framework of which can be applied to numerous other settings. Our team has utilized a virtual case presentation series that can be accessed remotely, the benefits of which have been previously studied.^7^ We use educational QI methodology with Plan-Do-Study-Act (PDSA) cycles with stakeholder input to continually evaluate and improve this partnership.

### Solution

The CHOP/LHD partnership began in November 2020, led by a paediatric haematologist and chair of paediatrics at LHD and two first-year paediatrics residents at CHOP, supported by CHOP faculty and a citizen diplomat. The learning activities are virtual case conferences, which occur every six to eight weeks via BlueJeans, a free virtual platform. Conferences are one hour and present an inpatient paediatrics case followed by group discussion. The cases are presented in English, with French translation provided throughout for Cameroonian participants. At the start of the partnership, stakeholders shared details regarding site-specific medical training, practice, and epidemiology. Roles and learning objectives were also defined. Project leaders have aimed to build an education partnership that is equitable, mutually beneficial, and sustainable. Primary drivers of this aim include shared leadership, goal setting, clear communication, and bidirectional learning.^8^ Therefore, our team uses iterative PDSA cycles to enact key interventions based on these drivers.

**Figure 1:**
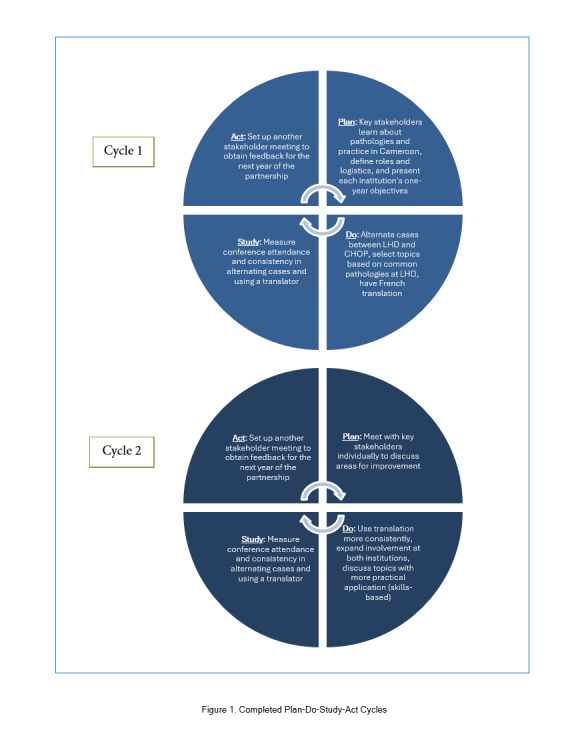
Completed Plan-Do-Study-Act Cycles

The initial PDSA cycle (November 2020-June 2021) focused on the shared objective of bidirectional knowledge exchange between trainees of all levels by addressing common pathologies at both hospitals and alternating the presenting institution. The second cycle (July 2021-August 2022) focused on the consistency of English-to-French translation and incorporating more skills-based sessions. The current cycle seeks to engage more trainees from LHD and enhance the utility of presentations for clinicians at both hospitals. Each PDSA cycle begins with a meeting between leaders from both institutions to evaluate progress towards our aim of creating an equitable, mutually beneficial, and sustainable partnership (Figure 1). Leaders have also incorporated WhatsApp, a free mobile application, to communicate about scheduling and gain regular, informal feedback. As a proxy for equitable engagement and sustainability, attendance has been our primary outcome measure. Consistency of French translation is our main process measure, fostering equity and mutual benefit. This QI educational initiative was reviewed and determined to not meet the criteria for human subjects' research by the CHOP Institutional Review Board.

### Evaluation

Over 24 months, participants from LHD/CHOP have presented 14 cases, 7 cases each (Table 1). Topics included febrile seizures, bacterial meningitis, and bronchiolitis, pathologies seen at both hospitals. Despite occasional technological difficulties such as poor internet connectivity, interventions in the first two PDSA cycles have resulted in fewer delays and more consistent interpretation. Specifically, we introduced an agenda with assigned roles and limited presentations to 30 minutes in order to troubleshoot technical difficulties and allow time for regular English to French translation. During PDSA cycle 2, we also incorporated practical sessions about bubble CPAP for respiratory support, a topic of interest for paediatricians at LHD. In our current PDSA cycle, we have selected conference times that allow more LHD physicians to attend and encouraged leaders from LHD to disseminate the conference information and BlueJeans link throughout their institution. We also incorporated post-conference surveys in January 2023 to better assess conference acceptability and utility.

**Figure 2:**
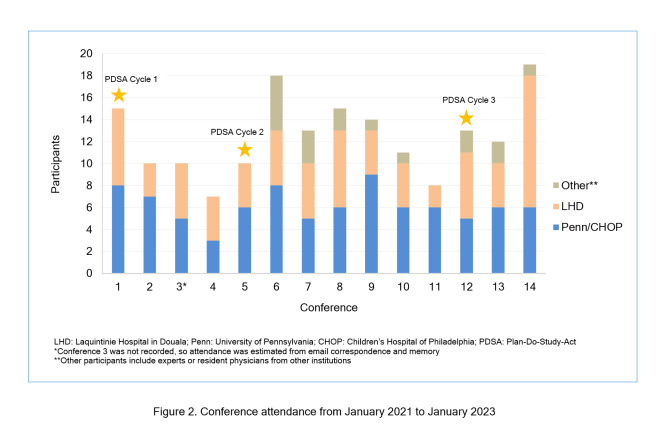
Conference attendance from January 2021 to January 2023

Over three PDSA cycles, we have observed overall equal attendance from LHD and CHOP. Further, at least one partnership leader from both institutions has been present at each conference. There has been an increase in attendance from both institutions, as well as the involvement of paediatricians from other hospitals worldwide (Figure 2). Anecdotal data from informal conversations has shown that colleagues from CHOP and LHD have appreciated the level of engagement from both institutions and have found the cases and discussions engaging. Preliminary post-conference survey results from our two most recent conferences (8 respondents total, four from each institution) show that 76% of respondents felt the presentation quality was very good/excellent, and 88% stated the conferences would be useful to their practice. Regarding translator use, 75% of respondents rated the consistency of translator use as good/excellent.

**Table 1:** Case conference topics

No	Date	Presenter Affiliation	**Topic**
1	Jan 2021	LHD	**Febrile Seizures**
2	Feb 2021	CHOP	**Paediatric Pulmonary Tuberculosis**
3	Mar 2021	LHD	**Graves' Disease**
4	May 2021	CHOP	**Bacterial Meningitis in Neonate**
5	Jul 2021	LHD	**Bronchiolitis**
6	Aug 2021	Other/CHOP	**Bubble CPAP in Low Resource Settings**
7	Nov 2021	LHD	**Feasibility of Bubble CPAP in LHD**
8	Jan 2022	CHOP	**Ehrlichiosis**
9	Mar 2022	LHD	**Stevens-Johnson Syndrome**
10	Apr 2022	CHOP	**Macrophage Activation Syndrome**
11	Jul 2022	LHD	**Near Drowning**
12	Sep 2022	CHOP	**Severe Acute Malnutrition**
13	Nov 2022	LHD	**DVT/Septic Thrombosis**
14	Jan 2023	CHOP	**Dissemination Bartonella/ Osteomyelitis**

LHD: Laquintinie Hospital in Douala; Penn: University of Pennsylvania; CHOP: Children’s Hospital of Philadelphia; PDSA: Plan-Do-Study-Act *Conference 3 was not recorded, so attendance was estimated from email correspondence and memory **Other participants include experts or resident physicians from other institutions

### Implications

We demonstrate an approach to virtual GHE that has fostered an equitable, mutually beneficial, and sustainable partnership over three years between paediatric programs in the United States and Cameroon. Challenges have included language barriers, time differences, and technological difficulties (e.g., internet and video streaming capacity). However, we highlight potential strategies to overcome barriers, such as consistent translator availability, committed and engaged leadership, and constant evaluation and improvement. To further enhance this partnership, we will continue using regular bi-directional WhatsApp communication, an annual goal-review meeting, and data from post-conference surveys. Others can replicate our approach to GHE partnerships with a focus on shared objectives and iteratively fostering equitable engagement.

## Acknowledgement

Thank you to the Children's Hospital of Philadelphia (CHOP) Global Health Centre for supporting and disseminating this partnership. Thank you to the mentors and guest consultants who contributed to the various case discussions.

## Conflict of Interest

The authors declare that they have no conflict of interest.
